# Laser Scanning In Vivo Confocal Microscopy of Clear Grafts after Penetrating Keratoplasty

**DOI:** 10.1155/2016/5159746

**Published:** 2016-02-29

**Authors:** Dai Wang, Peng Song, Shuting Wang, Dapeng Sun, Yuexin Wang, Yangyang Zhang, Hua Gao

**Affiliations:** ^1^Shandong Eye Hospital, Shandong Eye Institute, Shandong Academy of Medical Sciences, Jinan 250012, China; ^2^Qingdao Eye Hospital, Shandong Eye Institute, Shandong Academy of Medical Sciences, Qingdao 266071, China

## Abstract

*Purpose.* To evaluate the changes of keratocytes and dendritic cells in the central clear graft by laser scanning in vivo confocal microscopy after penetrating keratoplasty (PK).* Methods.* Thirty adult subjects receiving PK at Shandong Eye Institute and with clear grafts and no sign of immune rejection after surgery were recruited into this study, and 10 healthy adults were controls. The keratocytes and dendritic cells in the central graft were evaluated by laser scanning confocal microscopy, as well as epithelium cells, keratocytes, corneal endothelium cells, and corneal nerves (especially subepithelial plexus nerves).* Results.* Median density of subepithelial plexus nerves, keratocyte density in each layer of the stroma, and density of corneal endothelium cells were all lower in clear grafts than in controls. The dendritic cells of five (16.7%) patients were active in Bowman's membrane and stromal membrane of the graft after PK.* Conclusions.* Activated dendritic cells and Langerhans cells could be detected in some of the clear grafts, which indicated that the subclinical stress of immune reaction took part in the chronic injury of the clear graft after PK, even when there was no clinical rejection episode.

## 1. Introduction

Corneal transplantation has a long history of more than 100 years [1, 2]. Microscopic technology has greatly improved the success rate of keratoplasty [3]. Clinical application of immunosuppressive agents, such as cyclosporine A and FK506, significantly reduces the frequency of acute rejection [3–9]. Even so, clinically, the corneal grafts have been found to display gradual functional deterioration for months to years after transplantation. That is, with no history of rejection, the corneal grafts eventually become edematous and opaque. This behavior has been attributed to progressive late endothelium failure or chronic corneal allograft dysfunction [10, 11], which may represent the leading cause of poor long-term survival rates after penetrating keratoplasty (PK). It was reported that the loss rate of endothelial cells was 0.6% per year in normal human corneas. The loss rate of endothelial cells in the graft was up to 4.2% per year, even when there was no rejection episode after surgery [12].

To date, few studies have been reported about chronic damage to the graft because of lack of species of the clear allografts clinically. We have no idea whether the clear graft with no sign of immune rejection suffers chronic immune damage. It is also controversial whether immunosuppressive agents can be used in these patients. Confocal microscopy is a noninvasive technique for investigations of the cellular structure of corneal physiology and disease. It offers visualization of the living tissues and provides greyscale images with greatly increased resolutions over light biomicroscopy and biocytology, which can observe ultrastructure of the cornea [13–15]. Due to the advantage of in vivo confocal microscopy, it is possible to observe the graft in vivo and activation of keratocytes. In this study, we observed the clear graft without any sign of immune rejection after PK by confocal microscopy and investigated whether immune cells and other keratocytes were involved in the chronic damage to corneal grafts.

## 2. Methods

### 2.1. Subjects

Patients who underwent PK at Shandong Eye Institute between November 1, 1997, and December 21, 2013, were examined by using slit-lamp biomicroscopy to determine whether the graft was clear. Grafts suffering any clinical immune rejection episode were excluded from the study. Thirty patients (30 eyes) were included. The mean age was 47.56 ± 13.10 years (range, 16 to 65 years). The mean preoperative uncorrected visual acuity (Log MAR) was 1.736 ± 0.48 (range, 1.00 to 3.00).

### 2.2. Groups

Thirty clear grafts were examined at 12.8 ± 8.7 years (range, 1–17 years) after surgery. Preoperative indications for keratoplasty were corneal ulcer (18 eyes), granular corneal dystrophy (three eyes), pseudophakic bullous keratopathy due to the anterior chamber lens (two eyes), corneal scar due to eye injury (two eyes), keratoconus (two eyes), and others (three eyes). Ten normal corneas of 10 subjects who were examined between 2009 and 2014 were used as concurrent controls for keratocyte density and subbasal nerve density. The mean age was 30 ± 4.8 years (range, 24 to 34 years). The mean uncorrected visual acuity was 1.776 ± 0.67 (range, 0.52 to 3.00).

### 2.3. In Vivo Scanning Confocal Microscopy of Corneas

Laser scanning in vivo confocal microscopy was performed in all subjects with the Heidelberg Retina Tomograph II Rostock Corneal Module (Heidelberg Engineering GmBH, Heidelberg, Germany). All eyes were anesthetized with a drop of 0.4% benoxinate hydrochloride (Chauvin Pharmaceuticals, Surrey, UK). Viscotears (Carbomer 980, 0.2%; Novartis, North Ryde, NSW, Australia) was used as a coupling agent between the applanate lens cap and the cornea. During the examination, all subjects were asked to fixate on a distance target aligned to enable examination of the central cornea. The central corneal thickness may increase with time after PK [16, 17]. For brevity, we referred to this variable as “the number of keratocytes.” Three randomly chosen images per subject were analyzed and statistically compared.

### 2.4. Records of Digital Images

The objective was adjusted to provide an en face view of the central cornea. The patient fixated on a target with the contralateral eye to minimize eye movements. Digital images of the central cornea were recorded with the optical section advancing through the full-thickness cornea. Each image represented a coronal section of the cornea that was approximately 380 *μ*m (horizontal) × 380 *μ*m (vertical) [18, 19]. The full thickness of the central cornea or the area within the central 2 mm diameter was scanned using the “section mode” of the device. This mode enables instantaneous imaging of a single area of the cornea at a desired depth. A “through focus” series of images of one cornea constituted one “scan” with more than 450 video frames, depending on the thickness of the cornea. Two to four scans which contained the clearest images of the central basal epithelium were acquired per eye.

### 2.5. Image Analysis

For each confocal microscopic examination, three images were taken from each of the following levels: subepithelial nerve plexus, anterior stroma, posterior stroma, and endothelium. The best-quality scan without motion artifact was selected for each cornea by an experienced observer (SW). For the density measurement, the cornea was divided into 3 layers: epithelium, stroma (anterior corneal stromal cells and posterior corneal stromal cells), and endothelium. The nuclei of the keratocytes sharply demarcated only the highly reflective ones, and the reflective keratocytes were visualized on examination of the stroma. Confocal microscopy permitted in vivo evaluation of Langerhans cells (LCs) and other immune cells within the human cornea, with a particular emphasis on cell morphology distribution in Bowman's membrane and stroma. The density of subepithelial plexus nerves was evaluated using NeuronJ, a free semiautomatic image analysis program, and ImageJ (http://www.imagescience.org/meijering/software/neuronj/; accessed November 2012), a plug-in to the program. Images of keratocytes were analyzed using a custom automated program, which objectively identified bright objects (presumed to represent keratocyte nuclei) and calculated keratocyte density [10, 20]. Ten percent of all images were recounted by one of the examiners (DS) to determine the interexaminer limit of agreement.

### 2.6. Statistical Analysis

Mean keratocyte density of the full-thickness stroma, keratocyte density for each layer of the stroma, number of keratocytes, and subbasal nerve density were compared between clear grafts after PK and controls. Differences were analyzed using unpaired *t*-tests if the data were distributed normally or using Wilcoxon rank sum tests if the data were not distributed normally. *P* ≤ 0.05 was considered statistically significant. Correlations between keratocyte density or subbasal nerve density and time after keratoplasty were assessed using Pearson correlation coefficients if the data were distributed normally or using Spearman tests if the data were not distributed normally. The annual rate of keratocyte loss (percentage of decrease per year) was calculated from the number of keratocytes at each examination and the interval between examinations by assuming that the number of keratocytes decreased as a simple first-order loss.

### 2.7. Medical Ethics

The research adhered to the tenets of the Declaration of Helsinki. Informed, written consent was obtained from all subjects after explanation of the nature and possible consequences of the study. The protocol used was approved by the ethics committee of Shandong Eye Institute.

## 3. Results

### 3.1. Evaluation of Corneal Nerves

**Figure 1:**
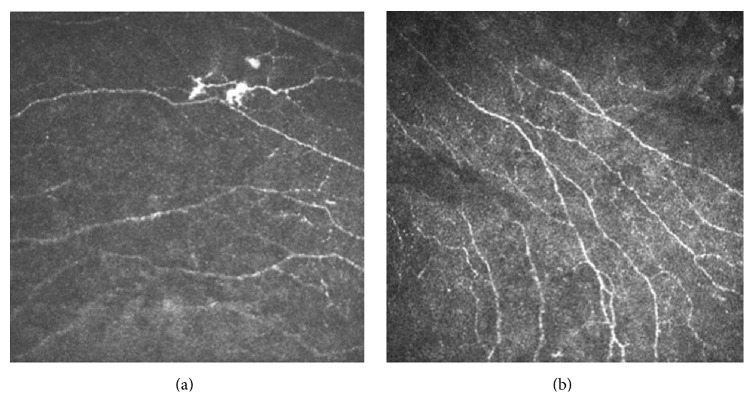
Laser scanning in vivo confocal microscopy images of (a) the subepithelial nerves in the patient after penetrating keratoplasty (regenerated in a random and disordered pattern) and (b) the subepithelial nerves of the healthy cornea (parallel neural contorts and regular morphology). (a) Scanning-depth: 45 *μ*m; age: 43 years. (b) Scanning-depth: 50 *μ*m; age: 29 years.

Mean density of subepithelial plexus nerves was lower in clear grafts (1506 *μ*m/mm^2^; range, 782–5846 *μ*m/mm^2^) (*n* = 30) than in controls (7020 *μ*m/mm^2^; range, 2371–12448 *μ*m/mm^2^; *P* ≤ 0.001) (*n* = 10). The subepithelial nerves regenerated smaller and shorter than the normal after PK in a random and disordered pattern (Figure 1).

### 3.2. Evaluation of Keratocytes

**Figure 2:**
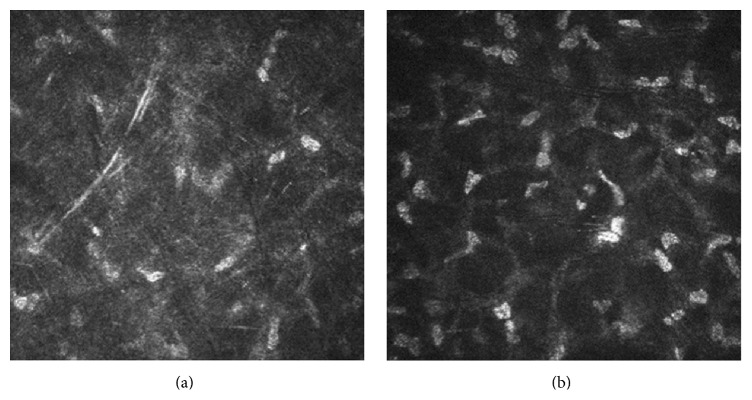
Laser scanning in vivo confocal microscopy images of (a) the stromal cells (disorderly and unsystematic) in the patient after penetrating keratoplasty and (b) the stromal cells in the healthy cornea. (a) Scanning-depth: 379 *μ*m; age: 44 years. (b) Scanning-depth: 401 *μ*m; age: 35 years.

Keratocyte density in each layer of the stroma was lower in clear grafts compared with controls. The anterior stromal cell density was 860.6 ± 299 cells/mm^2^ (range, 493–1404 cells/mm^2^) in clear grafts, while it was 1099 ± 164.9 cells/mm^2^ (range, 798–1380 cells/mm^2^) in normal corneas (*P* = 0.018). The posterior stromal cell density was 607.6 ± 263 cells/mm^2^ (range, 295–1203 cells/mm^2^) in clear grafts, significantly lower than that in normal corneas (754.9 ± 90.37 cells/mm^2^; range, 587–1380 cells/mm^2^; *P* = 0.024). The shape of a few nuclei transformed from the ellipse to the irregular spindle. The stromal cells were disorderly and unsystematic in patients after PK, and some cells became scars (Figure 2).

### 3.3. Evaluation of Endothelium Cells

**Figure 3:**
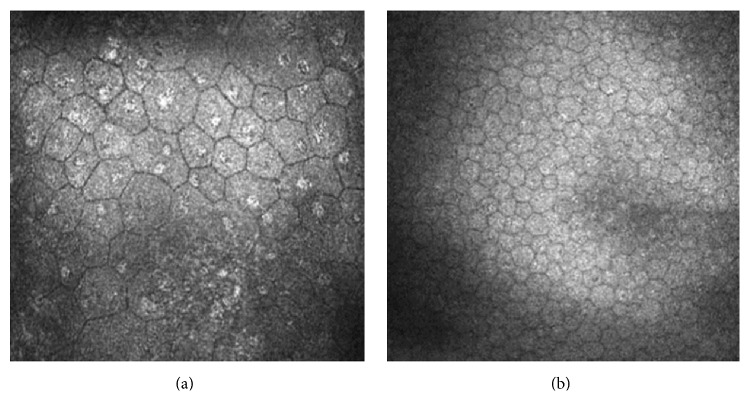
Laser scanning in vivo confocal microscopy images of (a) the corneal endothelium cells (heptagon) in the patient after penetrating keratoplasty and (b) the corneal endothelium cells in the healthy cornea. (a) Scanning-depth: 453 *μ*m; age: 39 years. (b) Scanning-depth: 460 *μ*m; age: 35 years.

Corneal endothelium cell density was 1561 ± 864 cells/mm^2^ (range, 480–3371 cells/mm^2^) in clear grafts, significantly lower than that in normal corneas (3099 ± 489 cells/mm^2^; range, 2798–3972 cells/mm^2^; *P* = 0.003). The endothelium cells changed from the hexagon shape into the heptagon or even more, with a bigger size. Some of the nuclei had high reflection and a few multinuclear cells. Endothelial cell nuclei of the PKs affected the brighter refraction of light than the normal (Figure 3).

### 3.4. Evaluation of Inflammatory Cells

**Figure 4:**
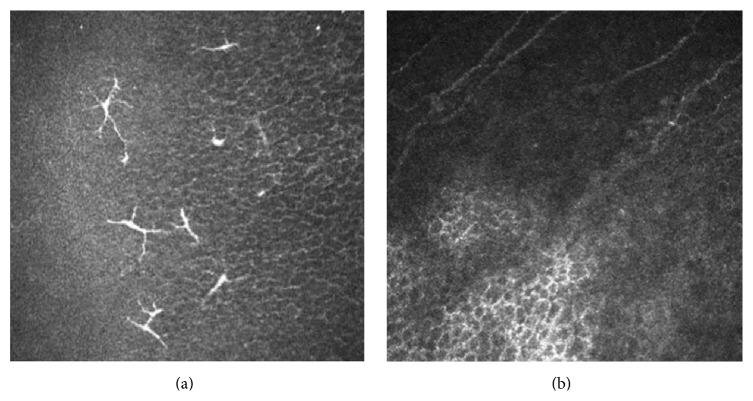
Laser scanning in vivo confocal microscopy images of (a) the Langerhans cells (white objects of split ends) activated in Bowman's membrane and stromal membrane in the patient after penetrating keratoplasty and (b) the same position in the healthy cornea. (a) Scanning-depth: 53 *μ*m; age: 51 years. (b) Scanning-depth: 49 *μ*m; age: 29 years.

Mean keratocyte density correlated weakly with time after surgery (*r* = −0.20; *P* = 0.05). The dentritic cells of five (16.7%) adults were active in Bowman's membrane and stromal membrane of the graft after PK. There were five or six activated LCs (white objects of split ends) in each scan of confocal microscopy (Figure 4).

## 4. Discussion

PK is one of the most important treatments for corneal disease. Every year about 100,000 people around the world accept this surgery for vision rehabilitation. However, chronic corneal allograft dysfunction or late graft failure after PK is a great problem threatening the long-term graft survival. The half time for the component was 21 years after PK according to a recent report [20]. Transplants can maintain about 20 years of transparency [12]. Although the causes of chronic corneal dysfunction are not very clear at present, literature and experimental data show that it has a certain relationship with cellular immune. After the graft is observed to be clear without any sign of immune rejection by slit-lamp microscopy, the results of confocal microscopy may be ignored. Confocal microscopy was used in our study to observe pathomorphological changes of clear central corneal grafts after PK for objective evaluation of graft changes.

Considering the neurological aspect, we mainly focused on subepithelial plexus nerves. Corneal nerves have an important function of ocular surface feeling and can also release all kinds of nutrients, like substance P and pituitary adenylate cyclase activating polypeptide, which promote the steady state of epithelium, and activation of tear secretion and blink reflex, so as to maintain the integrity of the structure and functional role of the ocular surface [21, 22]. Moreover, corneal nerves can promote corneal epithelial cell proliferation and migration and adjust the differentiation and proliferation of stem cells of corneal limbus by conducting the growth factor signals-*β* and completing repair of the graft [23]. It has been proved that nerve regeneration is very slow after PK no matter in what species, and the sensation of the cornea is difficult to fully recover. The transplanted cornea nerve rupture may cause the slow regeneration. Regeneration of postoperative matrix is incomplete, and the sensitivity of the corneal central nerve is closely correlated with the morphology and density of the substrate nerve [24, 25]. The short nerves may produce fewer neuropeptides, leading to the random pattern.

Normal corneal stromal cells are stable and do not differentiate or proliferate. The number of stromal cells was remarkably decreased after keratoplasty in our series, suggesting an instable state. We supposed some stromal cells may be removed by the immune cells. Stromal haze may be an important phenomenon of keratocytes, corresponding to accumulation of amorphous acellular reflective structures and the results of fibrosis. Stromal cells are the main components of normal corneal stroma, responsible for the synthesis of collagens and extracellular matrix and playing a quite important role in maintaining corneal transparency [26]. When amorphous acellular reflective materials are reduced, the recognizable stromal cells are gradually increased. During the regenerative repair of cornea, stromal cells also can enter the corneal injury by proliferative migration instead of transforming to repair-type fibroblasts [27, 28]. The stromal scar and the changing shapes of nuclei may hint stromal cells in the “state of stress.”

Corneal endothelium is the most important layer of the graft. In our study, when the number of endothelial cells declined, corneal edema and opacification occurred and affected the visual quality. This was consistent with the results of the study by Bourne [17]. Chronic corneal allograft dysfunction is mainly characterized by the loss of endothelial cells and changes in the morphology of endothelial cells. But no obvious immune rejection was found in our patients. Patel et al. [18] performed confocal microscopy to examine the cornea in 505 patients with different eye diseases. They found that among 17 patients treated by PK, reflection of endothelial nuclei can be observed in 9 patients (53%), and endothelial cells declined faster than the physiological speed [29]. It was reported that the defected cell area was mainly filled with migrated and enlarged cells from the surrounding area [29]. Some investigators speculated that the increased area of endothelial cells or stretching of the cells would lead to a thinner endothelial cell layer and fewer contents of cytoplasm per unit area. This is a kind of compensatory mechanism of cells. In endothelial cells with an increased area, reflection of dual cores and even multiple cores appeared in our series. This may be a phenomenon of cell fusion. Morphological changes of corneal endothelial cell nuclei may affect the refraction of light, making bright reflection of endothelial nucleus be directly observed under a confocal microscope [27, 30]. These phenomena may also hint the graft after PK in a state of stress.

Both the occurrence time of immune rejection after corneal transplantation and whether the immune cells are involved in the rejection remain controversial. Corneas are allosomes with good histocompatibility. It seems impossible if there is no presence of immune cells. However, we did not find a large number of immune cells or inflammatory cells in the patients at the end stage of retransplantation by ultrastructure observation of the corneal transplant. Therefore, detection of the immune cells in vivo had great significance in guiding the clinical medication. We noticed that not all grafts were adhered with activated immune cells (especially LCs). The results were roughly consistent with the previous reports [11, 28, 31]. Apparent immune cells were found in only 16% of the patients, suggesting that some patients were still affected. Immunosuppressants are routinely applied in our hospital after PK. Patients with a regular follow-up are given low concentrations of corticosteroid eye drops or immune inhibitors, which may reduce the number of immune cells in the grafts. Active immune cells invade the corneal graft bed from the vascular network of corneoscleral limbus and migrate to the corneal graft [32]. The decreasing number of corneal nerves and keratocytes indicates the graft may have chronic inflammation and be in the state of stress. These immune cells are not enough to cause a clinical visible immune rejection episode, but they may cause chronic damage to corneal grafts and gradually decrease the cell function. Adhesion mechanism of immune cells has not been clearly identified, and further investigations are needed. In our study, the patients were scheduled to follow-up every three months. But some patients failed to present in time. Therefore, data were not complete.

In summary, the subepithelial plexus nerves, keratocytes, and endothelial cells may significantly decline in number in the central clear graft after PK. The graft after PK is in a state of stress, affecting the normal physiological function of keratocytes and leading to the declination of graft function. Immune factors and other nonspecific factors are involved in the development of chronic dysfunction of the corneal graft.
